# Association between kinking of the cervical carotid or vertebral artery and ischemic stroke/TIA

**DOI:** 10.3389/fneur.2022.1008328

**Published:** 2022-09-13

**Authors:** Junjie Wang, Jun Lu, Peng Qi, Chunwei Li, Ximeng Yang, Kunpeng Chen, Daming Wang

**Affiliations:** ^1^Department of Neurosurgery, Beijing Hospital, National Center of Gerontology, Beijing, China; ^2^Institute of Geriatric Medicine, Chinese Academy of Medical Sciences, Beijing, China

**Keywords:** stroke, transient ischemic attack, carotid artery, vertebral artery, kinking

## Abstract

**Introduction:**

Kinking of the cervical carotid or vertebral artery is a common structural abnormality in patients with cerebrovascular disease. However, there is no consensus about the relationship between kinking and ischemic stroke/TIA. We aim to determine the effect of arterial kinking on ischemic stroke/TIA.

**Methods:**

A retrospective study was performed on patients who underwent cerebral angiography with DSA between January 2014 and December 2018. Demographic information and comorbidities were recorded. Each anatomical circulation system was defined as an observation unit. Kinking and stenosis of each circulation unit were recorded. Ischemia stroke or TIA within 6 months and its location were assessed as an outcome. Logistic regression with a generalized estimating equation approach was used for the analysis.

**Results:**

A total of 1,062 patients (mean age 57.9 ± 14.5 years, 740 males and 322 females) were included in the study. Of the patients, 369 (35%) had kinking and 771 (73%) had ischemic stroke/TIA. There were 110 left anterior, 90 right anterior, and 308 posterior circulation units, among which 343 had mild, 160 had moderate, and 243 had severe kinking. Multivariate regression analysis showed that ischemic stroke/TIA was associated with severe kinking (OR 1.39, 95% CI 1.03–1.88, *P* = 0.03). Posterior circulation was more vulnerable to acute ischemia than left anterior and right anterior circulation (OR 3.58, 95% CI 2.81–4.56, *P* < 0.0001).

**Conclusion:**

Severe kinking of the cervical carotid or vertebral artery may be associated with a higher risk of ischemic stroke/TIA, especially when the kinking is located in the posterior circulation.

## Introduction

Stroke is the second most common cause of mortality and the third most common cause of disability worldwide (1). China accounts for the greatest burden of stroke in the world (2). In China, stroke is a major cause of mortality, and long-term physical and cognitive impairment (3). A population-based study shows that even in recent years (from 2013 to 2019), the weighted prevalence of stroke in China still increased significantly from 2.28 to 2.58% (4). Cervical artery disease has been generally regarded as an important cause of stroke (5); however, although there has been swift development in treating atherosclerotic disease with endarterectomy or arterial stenting in past decades, there has been less improvement in the understanding and treatment of non-atherosclerotic diseases.

**Figure 1:**
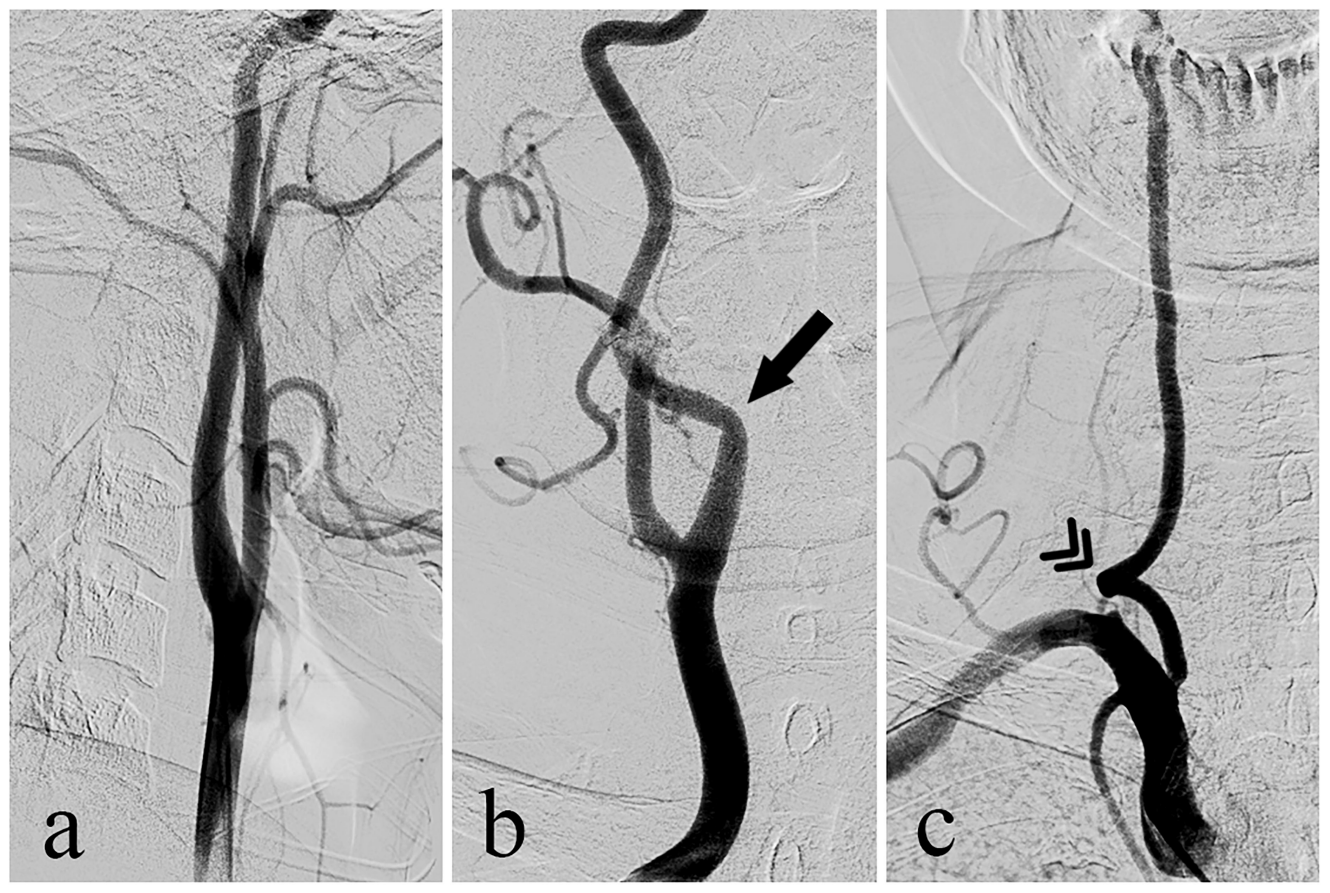
Diagram of DSA based on **(a)** normal course of carotid artery, **(b)** kinking of carotid artery, and **(c)** vertebral artery.

Kinking of the cervical carotid or vertebral artery (shown in Figure 1) is a common non-atherosclerotic structural abnormality in the elderly and patients with cerebrovascular disease (6–8). Cervical arterial kinking has been reported to be associated with hemodynamic impairment and an increased risk of stroke or TIA (9–13). However, previous findings are inconsistent (14, 15), owing to numerous confounding variables and small sample sizes. In addition to some common risk factors for cerebrovascular disease that need to be adjusted, a significant bias was related to the neglect of anatomical features of cerebral vessels. Cerebral circulation is naturally divided into three independent anatomic units (bilateral internal carotid systems and the vertebrobasilar system), and generally structural vascular lesions mainly involve their “downstream” territory, which leads to local neurological symptoms or signs. The outcomes of published studies were based on individual observations of ischemic events, which lacked accurate identification of the ischemic circulation unit and might indicate an incorrect relationship between ischemia of one circulation and lesions in another.

Thus, to address this limitation, we performed this large-scale study to fully assess the association between cervical arterial kinking and ischemic stroke/TIA.

## Materials and methods

### Study patients

**Figure 2:**
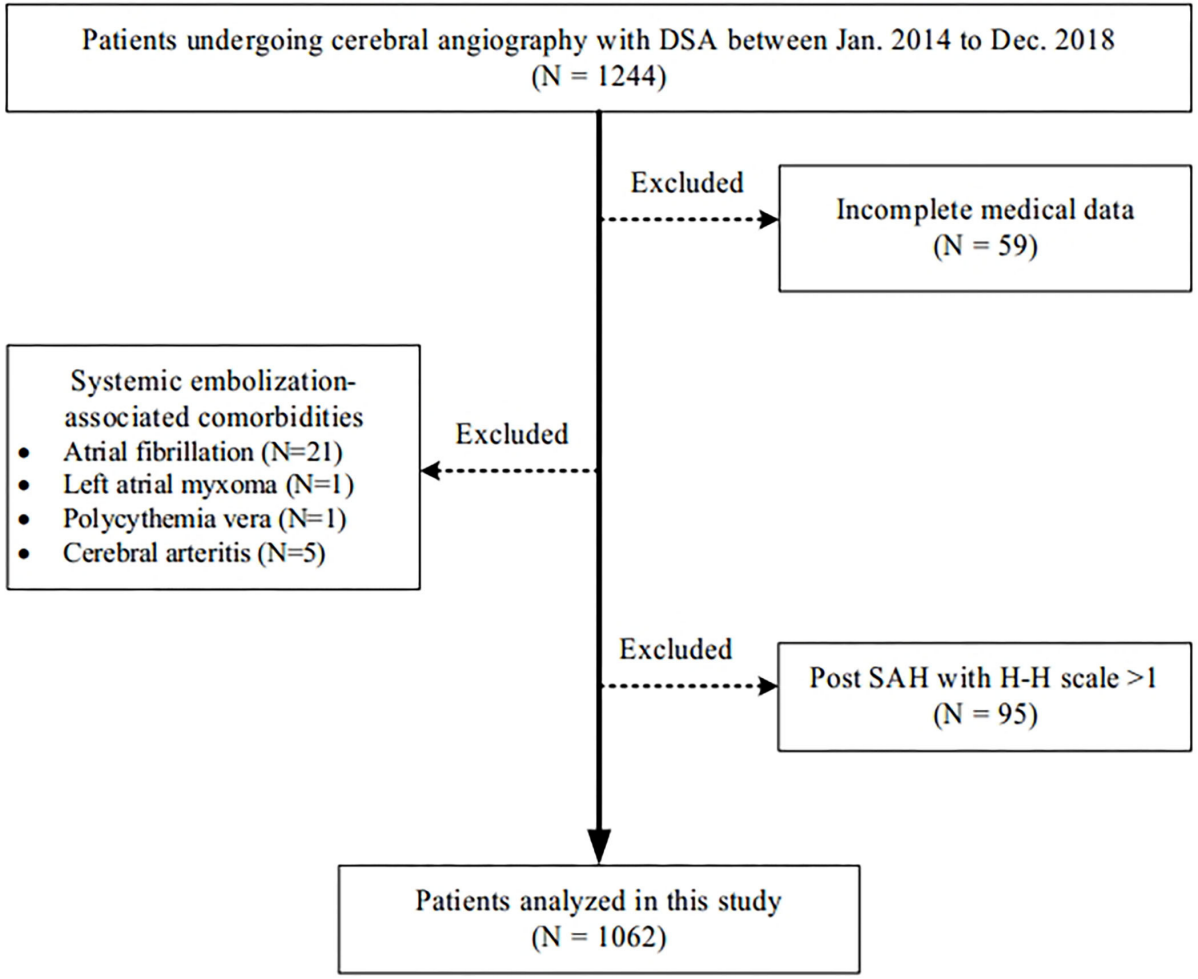
Flow chart of this retrospective study.

We retrospectively reviewed the data of 1,244 patients who underwent their first cerebral angiography with digital subtraction angiography (DSA) as a diagnostic or pretherapy procedure between January 2014 and December 2018 in our institution (Figure 2). Major exclusion criteria included systemic embolization-associated comorbidities (21 atrial fibrillation, 1 left atrial myxoma, 1 polycythemia vera, and 5 cerebral arteritis), post subarachnoid hemorrhage (SAH) with a Hunt-Hess scale >1 (unable to identify the presence and location of ischemic symptoms, *n* = 95), or incomplete medical data (*n* = 59). This study was approved by the Institutional Review Board (IRB) at Beijing Hospital (Approval No. 2019BJYYEC-130-01). The requirement for informed consent was waived because of the retrospective nature of the study.

### Kinking and stenosis measurement

All identifications and measurements of arterial kinking and stenosis were based on multiple views (including but not limited to standard posteroanterior and lateral projections) of cerebral DSA images.

**Figure 3:**
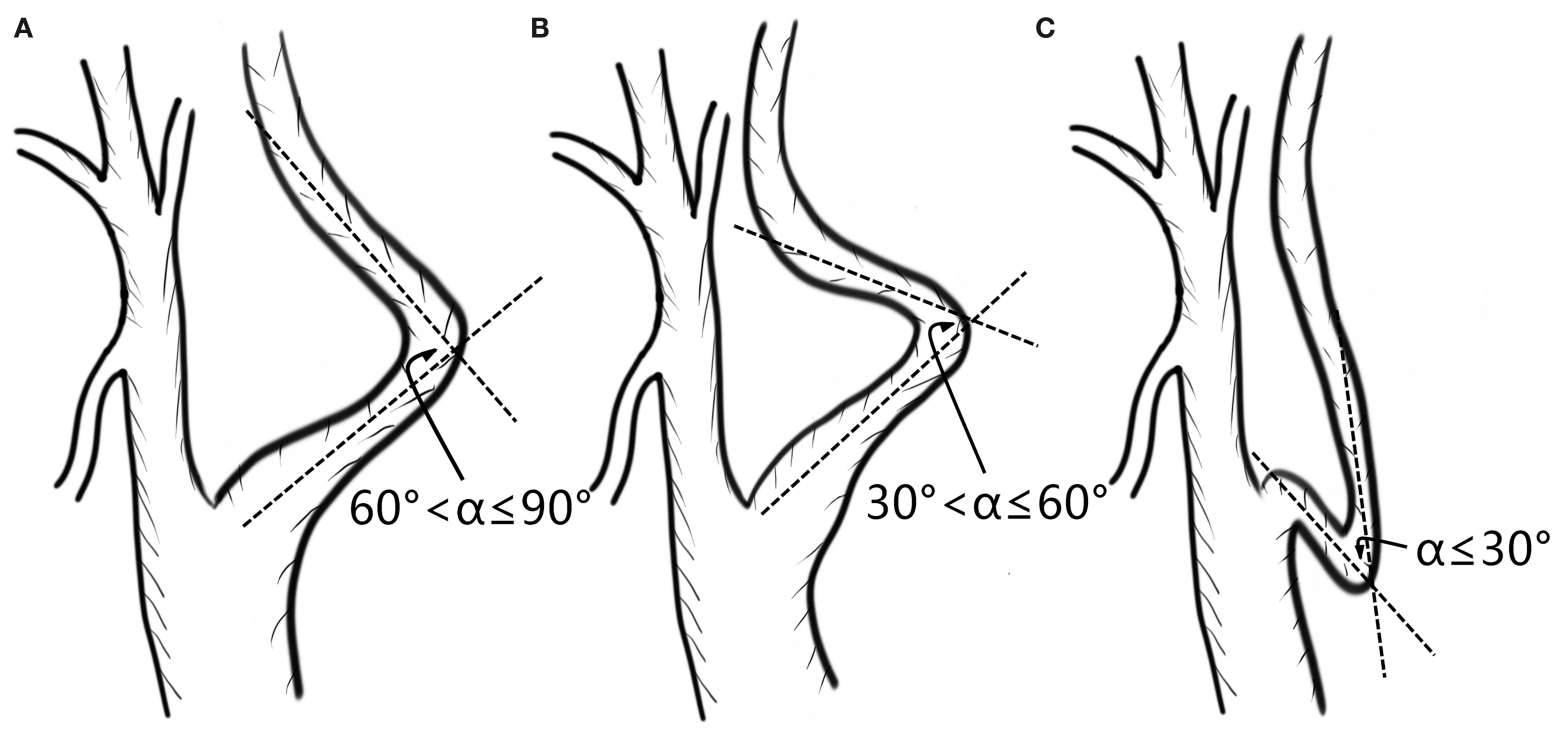
Classification of kinking according to the angle of curvature: mild **(A)**, moderate **(B)**, and severe **(C)**.

Kinking was classified according to the angle of curvature: mild (61°-90°), moderate (31°-60°), and severe (0–30° or circular configuration) (shown in Figure 3). According to the NASCET criteria, extracranial artery stenosis was graded as mild (0–49%), moderate (50–69%), or severe (70–99%). Intracranial artery stenosis was measured by the WASID method and divided into mild (0–49%) and severe (50–99%) grades.

Two neuroradiologists (Jun Lu and Peng Qi) who were blinded to the clinical history and the results of other diagnostic tests reviewed all DSA images (in the anterior–posterior and lateral standard views, with/without 3D rotational reconstruction) of included patients in parallel, and any discrepancies were resolved by consulting the senior doctor (Daming Wang). The presence and grade of kinking and stenosis at each circulation unit were recorded.

### Assessment of outcome

The major outcome was ischemic stroke or TIA in any circulation territory within 6 months before administration. For convenience, we collectively refer to ischemic stroke and TIA as acute cerebral ischemia (ACI). ACI was defined as an acute onset of neurologic dysfunction of any severity consistent with focal brain ischemia, with imaging confirmation of an acute vascular ischemic pathology or without any imaging confirmation of an acute vascular ischemic pathology, but imaging and clinical data do not suggest a non-ischemic etiology. The diagnosis and localization of ACI were conducted by two experienced neurologists based on all available information, including medical charts and imaging studies, except vasculature imaging data.

### Covariables

Patient-specific variables, including age, sex, and coexisting conditions, were extracted from medical records. Comorbidities consisted of hypertension, diabetes mellitus, coronary heart disease, hyperlipidemia, alcohol consumption, and smoking. Circulation-related variables included kinking and stenosis.

### Statistical analysis

Descriptive statistics were calculated for the entire study population and stratified according to patient group (ACI group and non-ACI group). Comparisons of covariates between the two groups were performed with the Student's *t*-test for continuous variables and the chi-square test for categorical variables. All statistical tests were two-sided, and *P* < 0.05 were considered to indicate statistical significance. The association between the covariates and the outcome was examined by using a multivariable logistic regression model, with a generalized estimating equation (GEE) approach and an exchangeable 3-by-3 correlation matrix. Because patient-specific variables apply common effects to every circulation unit, the outcomes at each circulation unit in the same patient were correlated. The GEE approach was implemented to account for the cluster that contained all circulation units from the same individual. The variable selection was performed by the stepwise method with an inclusion criterion of *P* < 0.05 to enter and *P* > 0.1 to exit. All analyses were conducted using the SAS software package (version 9.2; SAS Institute Inc., USA).

## Results

### Patients and circulation units

The final population included 1,062 patients (mean age 57.9 ± 14.5 years; 740 men and 322 women), with 2,885 circulation units (989 left anterior circulation units, 971 right anterior circulation units, and 925 posterior circulation units; 301 circulation units were excluded due to total occlusion of cervical segments).

Among the included patients, 771 (73%) suffered acute ischemic stroke/TIA within 6 months, 369 (35%) had at least 1 kinking in the cervical carotid or vertebral artery, 159 (15%) and 366 (34%) had extra-cranial and intra-cranial artery stenosis, respectively. In terms of the neurovascular risk factors, 642 (60%) had hypertension, 289 (27%) had diabetes mellitus, 191 (18%) had coronary heart disease, 360 (34%) had dyslipidemia, 197 (19%) had a history of ischemic stroke/TIA, 438 (41%) were smokers, and 255 (24%) were excessive drinkers.

Kinking was present in 508 circulation units of 369 patients, including 343 mild, 160 moderate, and 243 severe kinkings located at 110 left anterior circulation units, 90 right anterior circulation units, and 308 posterior circulation units.

### Predictor variables and demographic characteristics

The predictor variables and demographic characteristics of patients are shown in Table 1. Unsurprisingly, more stenotic lesions were located in patients in the ACI group (*P* < 0.0001). However, the presence of kinking did not significantly differ between groups (*P* = 0.21). Patients in the ACI group were older (*P* < 0.0001) and more likely to be male (*P* < 0.0001), smokers (*P* < 0.0001), and drinkers (*P* < 0.0001). These patients more frequently suffered from hypertension (*P* < 0.0001), diabetes mellitus (*P* < 0.0001), coronary heart disease (*P* < 0.0001), dyslipidemia (*P* < 0.0001), and historical ischemic stroke/TIA (*P* < 0.0001).

**Table 1:** Baseline demographics and predictor differences between study groups.

	**ACI^†^ group**	**Non-ACI^†^ group**	***P*-value**
No. of observations	771	291	–
Demographic			
Age	61.0 ± 12.5	49.9 ± 16.3	<0.0001
Male sex	76.8%	50.9%	<0.0001
DSA^‡^ imaging			
Kinking	28.7%	50.9%	<0.0001
Extracranial stenosis	71.1%	12.4%	<0.0001
Intracranial stenosis	29.2%	4.1%	<0.0001
Neurovascular risk factors			
Hypertension	69.3%	37.1%	<0.0001
Diabetes mellitus	34.2%	8.6%	<0.0001
Coronary heart disease	21.7%	8.2%	<0.0001
Dyslipidemia	41.5%	13.7%	<0.0001
History of ischemic stroke/TIA^§^	24.0%	4.1%	<0.0001
Cigarette smoking	46.4%	27.5%	<0.0001
Excessive alcohol consumption	28.9%	11.0%	<0.0001

^†^ACI, acute cerebral ischemia,^‡^DSA, digital subtraction angiography, ^§^TIA, transient ischemic attack.

### Kinking and acute cerebral ischemia

The results of a GEE model that simultaneously included all predictors and interactions between kinking and extracranial arterial stenosis are presented in Table 2. Severe kinking (OR 1.39, 95% CI 1.03–1.88, *P* = 0.030) was independently correlated with an additional 39% increase in the probability of ACI. Posterior circulation was more vulnerable to ischemia (OR 3.58, 95% CI 2.81–4.56, *P* < 0.0001). No significant interaction between kinking and arterial stenosis was found.

**Table 2:** Multivariable GEE model and adjusted odds ratios for acute cerebral ischemia.

**Factors**	**Adjusted OR**	**95% CI**	***P*-value**
Male	1.54	(1.31, 1.82)	<0.0001
≥65 years old	1.20	(1.04, 1.38)	0.0120
Hypertension	1.31	(1.13, 1.53)	0.0005
Diabetes mellitus	1.21	(1.05, 1.39)	0.0104
Dyslipidemia	1.35	(1.18, 1.55)	<0.0001
History of ischemic stroke/TIA	1.34	(1.14, 1.58)	0.0004
Extracranial arterial stenosis	2.01	(1.79, 2.26)	<0.0001
Intracranial arterial stenosis: ≥50%	8.17	(6.08, 10.98)	<0.0001
Posterior circulatory system	3.58	(2.81, 4.56)	<0.0001
Kinking			
Mild/moderate	1.01	(0.80, 1.28)	0.9174
Severe	1.39	(1.03, 1.88)	0.0301

## Discussion

Cervical arterial kinking is not an uncommon condition, especially in the elderly, with a prevalence of 22%−58%, according to previous literature (7, 8, 13, 15, 16). Theoretically, as a geometric change in arterial course, kinking could possibly contribute to cerebral ischemia in the following ways: (1) hemodynamic mechanism: energy loss due to turbulence at the site of kinking decreases the distal perfusion pressure; (2) thromboembolism mechanism: flow disturbance caused by vessel bending impairs local vascular intima, where a microembolus could form and lead to an embolic event; (3) lesions with extreme kinking might directly develop luminal stricture; and (4) in some cases, neck rotation could occlude the kinked artery temporarily.

Despite these hypotheses, there is still no consensus on the relationship between kinking and ischemic cerebrovascular disease. Some studies have suggested a positive correlation (6–8, 10, 11, 13). Some authors have even achieved the goal of preventing cerebral ischemia by eliminating kinking through surgery (12, 17). However, other studies obtained negative results (14, 15). Most of the previous publications were limited by a small sample size, which made it difficult to adjust for all covariates (e.g., age, diabetes mellitus) in multivariate analysis. In addition, the evaluation of kinking or stenotic lesions was performed by only ultrasound in some studies (7, 8, 14, 15), instead of DSA or computed tomography angiography (CTA), which are significantly more sensitive techniques. Furthermore, a quantitative or semiquantitative (graded) description of the degree of kinking was lacking in some papers (8, 9, 13).

Because a kinked lesion and an ischemic event might not be located in the same circulation unit, we separated the arteries supplying the brain and introduced the concept of the cerebral circulatory system as the observation unit. This simple model established a logical, direct connection between arterial lesions (stenosis or kinking) and ischemia in the feeding territory of the same arteries. To our knowledge, this was the first study to focus on each detailed culprit vessel in this research field.

In this large sample study (the largest in the past 50 years), after controlling for other risk factors, we confirmed that patients with severe kinking had an increased risk for ACI. The effect (OR = 1.39) of kinking was even greater than that of some conventional risk factors (hypertension, diabetes mellitus, and dyslipidemia). Our results provide important information for the prognosis of this common structural abnormality and could help clinicians in the early detection of patients at a higher risk for cerebral ischemia. Patients with severe kinking should receive more attention and follow-up for the possible risk of cerebral ischemia. The subsequent prospective studies might provide further clinical evidence for the prophylactic treatment of selective patients.

According to the analysis of each observation unit, kinking is more likely to lead to ischemia of the posterior circulation than in the other circulation units. This finding might be attributed to the anatomic features of the vertebrobasilar system, which has a notably thinner diameter than that of the anterior circulation. Therefore, the weak reserve capacity would easily lead to reduced blood flow at the distal tissue prior to decreased perfusion pressure by kinking or even mild stenosis. In addition, the extracranial vertebral artery goes into the transverse foramina, and its mobility is limited by the vertebrae. Neck movement may aggravate the existing kinking or stenosis of the vertebral artery.

Our study has several strengths, including its large sample size, standard evaluation method (DSA), and identification of ischemia events at each observation unit for the first time. However, there are some limitations to our studies. Our study was a retrospective single-center study; thus, the cases were not necessarily a representative sample of the general population. In particular, due to the long time span of the study cases, the traceability of medical records and the popularity of imaging examinations in the early years were limited, which might lead to misjudgment of outcomes (such as the etiology and type of stroke), and might cause some recall or collection bias. In addition, the classification of kinking was based on neuroradiologists, which is subject to their professional experiences. A prospective, multicenter study with accurate geometric methods consistent with hemodynamics (e.g., three-dimensional vascular parameters) may be necessary to improve the estimates.

## Conclusion

Kinking is not only a common phenomenon in the cervical carotid or vertebral artery course. Severe kinking may be associated with a higher risk of ischemic stroke/TIA, especially when the kinking is located in the posterior circulation.

## Data availability statement

The raw data supporting the conclusions of this article will be made available by the authors, without undue reservation.

## Ethics statement

The studies involving human participants were reviewed and approved by Ethics Committee of Beijing Hospital. The Ethics Committee waived the requirement of written informed consent for participation.

## Author contributions

JW: study design and collection, analysis, and interpretation of data. CL: data collection, manuscript preparation, and review. JL and PQ: analysis of images. XY and KC: data collection. DW: study concept and design, analysis of images, and critical revision of the manuscript for intellectual content. All authors contributed to the article and approved the submitted version.

## Funding

This research is supported by the Non-profit Central Research Institute Fund of Chinese Academy of Medical Sciences (No. 2019TX320002), Capital's Funds for Health Improvement and Research (2020-4-4053), and Beijing Hospital Clinical Research 121 Project (BJ-2018-086).

## Conflict of interest

The authors declare that the research was conducted in the absence of any commercial or financial relationships that could be construed as a potential conflict of interest.

## Publisher's note

All claims expressed in this article are solely those of the authors and do not necessarily represent those of their affiliated organizations, or those of the publisher, the editors and the reviewers. Any product that may be evaluated in this article, or claim that may be made by its manufacturer, is not guaranteed or endorsed by the publisher.
